# Social capital and well-being of the elderly ‘left-behind’ by their migrant children in India

**DOI:** 10.1186/s12889-023-17012-9

**Published:** 2023-11-09

**Authors:** Manoj Dakua, Ranjan Karmakar, Hemkhothang Lhungdim

**Affiliations:** 1 International Institute for Population Sciences, Mumbai, 400088 India; 2 Department of Public Health and Mortality studies, International Institute for Population Sciences, Mumbai, 400088 India

**Keywords:** Social capital, Depression, ADL, IADL, Migrants

## Abstract

**Background:**

The study aims to examine the association between individual forms of social capital and the well-being of the elderly ‘left-behind’ parents and to determine if there is a gender difference within the possible relationship.

**Methods:**

This study applied the first wave of the Longitudinal Ageing Study in India (LASI, 2017-18) data. In this study, the respondents were 4,736 older parents ‘left-behind’ by their migrant adult sons. We employed descriptive statistics and bivariate analysis to assess the study sample’s characteristics. The proportion test was performed to examine if there was a significant gender difference among older adults regarding depression, ADL, and IADL impairments. In addition, binary logistic regression was utilized to investigate the associations between social capital and elderly parents’ health outcomes.

**Results:**

This study found a significant gender difference in depression (male: 8.26%; female:11.32%; P < 0.001), ADL (male:20.23%; female:25.75%; P = 0.032), and IADL (male: 33.97% female: 54.13%; P < 0.001) limitations. Elderly parents who did not participate in any social activity had a higher odd of ADL (aOR: 2.44; 95%CI: 1.882–3.171; P = < 0.001) and IADL (aOR: 1.22; 95%CI: 1.034–1.766 ; P = < 0.001) limitations. Networking with friends through phone/email conversations has a substantial impact on lowering depression in older parents. Older adults with good personal social capital were less likely to have depression, ADL, and IADL limitations.

**Conclusion:**

Personal social capital is closely associated with the well-being of left-behind older parents. More efforts should be in place to increase the stock of social capital in this group with focused gender disparity.

## Background

Uneven economic and social development has emerged from globalization and industrialization, resulting in a significant increase in adult migrants. Various push factors (poor productivity, underemployment, inadequate income, inadequate non-agricultural occupations, inability to raise sufficient grains or crops etc.) in underserved areas have also played a key role in increasing adult migration [1, 2]. In third-world countries, the growing number of older people has resulted in a scarcity of institutional support that fails to satisfy their expectations [3, 4]. As a consequence of India’s changing demographics, older people now have a longer life expectancy and thus require more care and support to carry out their everyday chores [5].

In contrary to Western countries, multigenerational households are the traditional norm in India [6]. It is a traditional practice [6] for most older people to live with their married son and family [7], yet recent studies have revealed a decline in family support among older parents [8, 9]. One of the leading reasons for the loss of family support for elderly parents is adult migration [10–12]. Several studies have been carried out in both developed and developing nations to examine the impact of adult migration on elderly parents who stay behind at home. Studies have found that being “left behind” has a detrimental influence on the mental health of the elderly and an adverse effect on ageing, including social isolation, lack of social support, and cognitive decline [13, 14].

Older parents who live alone or with their spouse after their children have left home to live independently are referred to as “empty nesters” by the social scientists [15, 16]. Previous studies on the “empty nest syndrome” [17] in older parents found that they are more likely to report anxiety disorder [15], depression [18], and loneliness [19]. According to the study, ‘Empty nest’ older parents are also more likely to report poor health-related quality of life [19]. Numerous studies argued that adult-child migration has negatively impacted the elderly’s health. In China, older parents of migrant children consistently reported poorer health than older parents whose children have not migrated, and adults who have migrated for more extended periods have a more significant impact on the health of their older parents than adults who have migrated for a shorter period [20].

A recent study in India on children’s migration and lifestyle-related chronic disease of ‘left-behind’ older parents found that those older parents with at least one migrant son were more likely to report chronic diseases like hypertension, diabetes, and heart disease compared to those whose children did not migrate [21]. Besides, research findings from both developing and developed countries demonstrated that left-behind parents were more likely to report depressive symptoms [17, 22, 23], poorer cognition [17, 22], low level of life satisfaction [22], and poorer mental health [24–28]. On the contrary, some studies have explored that migrant children’s remittances positively impact the health of older adults who are left behind [29]. Remittance from children is connected to higher household income and a lower risk of depression among elderly parents who are left behind [30]. Furthermore, a multi-country study shows that the elderly with migrant children had more social capital, enhancing older adult’s Body Mass Index (BMI) and self-reported health [29]. This study focuses on “left-behind” older parents, applying the operational definition of “left-behind,“ used in prior studies, which is older parents reside in their country of origin or place of origin with one or more biological or adoptive emigrant children or out migrants [31, 32].

The link between social capital produced from the social contacts established in our daily lives and health outcomes has been extensively researched since the 1990s [33, 34].Several studies have demonstrated a positive impact of social capital in health outcomes [35–37]. In addition, the health disparity between men and women is often discussed [38, 39]. Men have a shorter lifespan than women; however, they have a healthier health assessment [40]. Women and men experience adult son migration differently– mothers experience more emotional support and fathers experience more practical assistance [41, 42]. Social capital also affects health differently [43]. Social networks can promote women’s preventive care, medication regimens, and healthier lifestyles, while men can engage in physical activities and healthy discussions. It has been widely accepted that there is also a gender divide in the relationship between social capital and health [44, 45]. However, no gender gap in this connection has been explored in India among older parents having at least one migrant adult son.

As a matter of interest, the current study is aimed to answer two questions in this regard. Firstly, what is the relationship between social capital and the health of elderly left parents? Secondly, is this possible relationship (if any) influenced by the gender of the parents?

### Definition and measurement of social capital

Social capital is controversial; no universally accepted definition or method exists to measure it [46–49]. It is difficult to describe one of the significant issues accounted for while defining social capital, whether it is an individual asset [49] or a communal asset [50]. Putnam’s definition of communitarianism social capital is more influential in public health, but different definitions of social capital have been developed [34]. Putnam defined social capital as “social structure,” emphasizing collaborative traits and the value of individual acts from the standpoint of cohesiveness [51, 52].

Trust, social networks, and social participation are commonly acknowledged as social capital indicators in the context of public health [34, 53]. The feeling of faith and anticipation that comes from thinking others will act softly and expectantly is what trust is all about [54]. It refers to one person’s belief or confidence in another person’s reliability, honesty, and integrity. Individual social contacts are social networks. It refers to the interactions and connections one develops with others personally and professionally. A stable system developed by social interactions and, involvement in various formal or informal activities is referred to as social participation [52]. It is also well known that social capital comprises cognitive and structural elements, with the past relating to trust and the latter to social networks and social partnerships [34, 52, 55]. In addition, social capital has been widely studied in two ways, i.e., bonding and bridging social capital [34]. Bonding means the internal social connection of individuals inside a homogeneous group, like caste groups and religious groups, in the Indian perspective. When an external social connection develops between members of heterogeneous groups, it is called bridging social capital [51, 52]. For this study, we have measured social capital among the elderly through trust, social networks, and social participation.

## Methods

### Data source

The first wave of the Longitudinal Ageing Study in India (LASI),which took place in 2017–2018, was applied for our study. LASI is a large-scale, nationally representative study of 72,250 older adults aged 45 and above to assess social, health, and economic well-being and the consequences of the Indian elderly. The survey used multistage stratified area probability sampling to arrive at the final numbers for people aged 45 and above and their spouses. The survey applied a three-stage sample design in rural regions and a four-stage sampling design in urban areas. The first step selected Primary sampling units, such as tehsils/talukas, and the second stage selected villages in rural and Wards in urban areas. Households were chosen in the third stage from rural areas, but there was an additional stage in urban areas. In the third stage, one Census Enumeration Block was chosen randomly, and households were selected from the CEB in the fourth stage. With four answer choices (inside village/city, inside/within the state, outside the state, and outside the country), the survey collected information about the place of living of each child of the parents who are currently not co-residing with parents. In addition, the survey gathered information on each child’s gender and age. Having an adult migrant son is defined in this study as having an adult son who lives outside of the state and country [56]. This study focuses on the elderly aged 60 and above with at least one living migrant adult son. The final sample size was 4,736 older adults aged 60 and above (male = 2,299; female = 2,437) after excluding parents without male children and parents with under 18 sons.

### Measures

#### Outcome variables

Health status, which encompassed physical and psychological well-being, was the outcome variable in this study. Activities of daily living [57] and instrumental activities of daily living [58] were included in physical health. Bathing, dressing, mobility, feeding, and toileting are the five indicators of Activities of Daily Life (ADL) [59]. ADLs were divided into two categories: “*No ADL limitation*” for elderly people who can perform all five tasks, and “*Having ADL limitation*” for elderly people who have trouble in completing any of them [60, 61]. Furthermore, Instrumental Activities of Daily Living (IADL) comprises seven activities: cooking and serving a hot meal, buying groceries, making phone calls, taking medications, doing housework or gardening, managing money (paying bills and keeping track of spending), and getting around or seeking an address in an unknown place [62]. Similarly, IADL difficulties were divided into two categories: “*No IADL limitation*” included those who could do all seven tasks, and “*Having IADL limitation*” included those who could not do any of them [60, 61]. The depression scale was utilized with three or more scores of 0 to 10 for CIDI-SF (Short-Form Composite International Diagnostic Interview) evaluations of psychological well-being [56]. It consists of ten questions with three or more positive responses assigned to “*diagnosed with depression*” [56]. This scale is internationally validated and comparable and has been field-tested and used in health surveys to diagnose probable major depression [62].

#### Main explanatory variables

The first wave of LASI survey gathered information on three components of social capital: trust, social participation, and social networks as bonding and bridging forms. In public health research, the bonding and bridging forms of social capital have been widely utilized [34]. A type of inner connectedness within persons uniform groups is called bonding social capital and bridging social capital relates to an external social link between individuals of diverse groups [52]. The first component, trust, was assessed by using the question below, *“With whom do you discuss the most of your personal matters?”* Responses from respondents were further categorized into ‘*having trust*’ and ‘*not having trust.’* People share their personal matters with others when they feel secure in their reliability. Participants were asked twelve questions to assess their social activities regarding *meetings or gatherings in clubs, organizations, or societies, eating outside, gaming, entertaining, visiting relatives or friends, using the digital platform, reading printed papers*, etc. We created a new dichotomous variable based on these twelve different responses; ‘*having any participation*’ and ‘*not having any participation*.’ Social networks were utilized as the study’s third component. The number of close friends determined the network size (ranging from 0 to 20)., The number of close friends was further divided into two categories; ‘having a close friend’ and ‘not having a close friend.’

Additionally, we evaluated the network density of individuals who had close friends separately based on their meetings and conversation frequency. Meetings with friends were classified as ‘not having meeting frequency’ (0) and ‘having any meeting frequency’ [1]. Talking with friends was also classified into two categories; ‘not having talking frequency’ (0) and ‘having talking frequency’ [1].

#### Socio demographic characteristics

Respondent age was grouped as 60–69 years (old), 70–79 years (old-old), and 80+ (oldest old). The sex of the respondents was categorized as male and female. The years of schooling were divided into four groups: 0 years, 1 to 5 years, 6 to 9 years, and 10 or more years. The living arrangement of the respondent was categorized as dichotomous form ‘living alone’ and ‘living not alone.’ Living not alone included living with spouse and/or others, with spouse and children, with children and others, and living with others only. ‘Currently in the union’ and ‘currently not in the union’ were the two categories used for marital status. Individuals who were currently married or in a live-in relationship were classified as ‘currently in the union,’ whereas those who were widowed, divorced, separated, deserted, or never married were classified as ‘currently not in the union.’ The current work status of the respondent was categorized as ‘currently working’ or ‘currently not working.’

#### Household characteristics

The monthly per capita consumption expenditure (MPCE) quintile was constructed using household consumption data. Eleven food-related and 29 non-food-related questions were asked to estimate the sample household expenditure. The reference period for food-related expenditure was seven days, whereas the non-food-related expenditure was 30 days and 365 days. MPCE was calculated using a summary measure of consumption and a standardized item reference period of 30 days [63]. The variable was categorized into five quintiles, from poorest to richest. The respondent’s religion was categorized as Hindu, Muslim, Christian, and Others. The place of the dwelling was classified as urban and rural.

### Statistical analysis

The study applied descriptive statistics and bivariate analysis to explore the health outcomes of ‘left behind’ elderly parents, The significance level differences in health outcomes (ADL, IADL, and Depression) among the elderly males and females ‘left behind’ were evaluated using the proportion test [64]. Furthermore, among the elderly left behind in India, binary logistic regression analysis was performed to examine the association between health outcomes (ADL, IADL, and Depression) and social capital, as well as other socioeconomic and demographic characteristics, were adjusted. Unadjusted and adjusted odds ratios (UOR and aOR) were present at 95% confidence intervals. STATA 14 was used for all statistical analyses.

## Results

### Socio-economic and demographic profile of left-behind older parents in India

Table 1 shows the socio-economic and demographic characteristics of 4,736 older parents who had at least one living migrant son, including 2,299 men and 2,437 women in the sample. Analysis indicates that 8.03% of Indian elderly females (n = 194) lacked trust in others, while 6.41% were males. About 5% of the male (n = 116) never participated in any social activity; on the one hand, 6.71% were females (n = 163). Around 64% of elderly males (n = 1490) and 79.40% of elderly females (n = 1935) did not have close friends. On the other hand, when we look at how many older males had met and talked with their friends, we observed that 34% (n = 796) had met and 19% (n = 441) had talked with friends, and counterparts were 20% (n = 491) and 7% (n = 191) respectively. A total of 10.53% (n = 242) of those males who took part were above the age of 80 and 9.31% (n = 227) were female, and roughly 46.04% (n = 1122) females of those who took part were currently single. Most of respondents (97.09% males and 93.3% females) were co-residing with family members and others. Regarding education, the majority of the female elderly (62.78%) had no formal education; while 34.45% of elderly males had ten years or more schooling. About 40% of older males were working, while 13.30% were females. The majority were from rural (60%) areas. Each of the MPCE quintiles had a substantially equal proportion of respondents.

**Table 1:** Background characteristics of older adult parent left behind by their migrant children, India, LASI (2017-18)

Background characteristics	Male (2299)		Female (2437)	
	n	%	n	%
Age				
60-69	1302	56.63	1521	62.41
70-79	755	32.84	689	28.27
80+	242	10.53	227	9.31
Year of schooling				
0	648	28.19	1530	62.78
1-5	441	19.18	400	16.41
6-9	418	18.18	210	8.68
10+	792	34.45	297	12.19
Currently working status				
Currently not working	1350	60.46	2113	86.70
Currently working	883	39.54	324	13.30
Marital status				
Currently in union	1951	84.86	1315	53.96
Not in union	348	15.14	1122	46.04
Living arrangement				
Alone	67	2.91	163	6.69
Not alone	2232	97.09	2274	93.31
MPCE quintile				
Poorest	345	15.01	386	15.84
Poorer	417	18.14	450	18.47
Middle	449	19.53	496	20.35
Richer	482	20.97	496	20.35
Richest	606	26.36	609	24.99
Religion				
Hindu	1696	73.77	1778	72.96
Muslim	290	12.61	335	13.75
Christian	199	8.66	216	8.86
Others	114	4.96	108	4.43
Place of residence				
Rural	1388	60.37	1444	59.25
Urban	911	39.63	993	40.75
Trust				
No trust on others	147	6.41	194	8.03
Having trust on others	2148	93.59	2223	91.97
Social participation				
No participation	116	5.06	163	6.71
Having participation	2175	94.94	2265	93.29
Friend				
No friend	1490	64.81	1935	79.40
Having friends	809	35.19	502	20.60
Meeting with friend				
No meeting frequency	1503	65.38	1943	79.83
Having meeting	796	34.62	491	20.17
Talking with friends				
No talking	1858	80.82	2244	92.16
Having talking with friends	441	19.18	191	7.84

Note- ‘n’ is not equal for all the variables because of missing cases

### Social capital and ADL

Table 2 represents the bivariate estimation of ADL limitation among older parents with at least one migrant son. ADL limitations were found to be more severe in older parents who lacked social activity, interpersonal trust, close friendships, meetings and conversations with their friends. Additionally, there is a significant gender gap in ADL limitation and social capital. Older males faced less ADL limitations (males 18.90; females 25.19 p = 0.015) and social participation (males 17.52; females 23.84 p = 0.021) than their female counterparts.

**Table 2:** Bivariate differences for ADL limitations and social capital among the older adult’s parents ‘left-behind’ by their migrant children, India, LASI (2017-18)

Variables	Have migrant son older adults			
	Male	Female	Z value	P value
Trust				
No trust	21.09	28.87	-0.79	0.429
Have Trust	18.90	25.19	-2.41	0.015
Social participation				
No participation	46.55	48.47	-0.21	0.827
Any participation	17.52	23.84	-2.31	0.021
Friend				
None	20.60	26.67	-1.96	0.049
Have friends	16.19	21.12	-0.97	0.330
Meeting with friends				
Have no meeting	20.56	26.66	-1.97	0.048
Have any meeting	16.21	20.98	-0.93	0.350
Talking with friends				
Have no talking	20.34	26.52	-2.19	0.028
Have any talking	13.61	13.61	0	1.000
Total	20.23	25.75	-2.04	0.032

Table 3 depicts the relationship between social capital and ADL limitation. Social participation, having close friends and conversing with them positively related to low-level ADL limitation even when other variables were adjusted. ADL limitations were 2.44 times (aOR: 2.44; CI: 1.882–3.171; p = < 0.001) higher for those who did not participate in any social activities, and 1.55 times (aOR: 1.55; CI: 1.142–2.118; p = < 0.001) higher for those who did not converse with their close friends. Additionally, there was a significant gender gap in the association between different forms of social capital and ADL limitation. The results indicate that males who did not participate in any social activities faced 2.96 times (aOR: 2.96; CI: 1.974–4.444; p = < 0.001) higher ADL limitation than their male counterparts, while females faced 2.17 times higher (aOR: 2.17; CI: 1.538–3.062; p = < 0.001) than their female counterparts. Social participation was equally important for both genders for ADL limitation. A close friend was considerably important for men for ADL. Along with having a close friend, women who did not communicate with their friend by mail or phone had 2.02 significantly (aOR: 2.02; CI: 1.212–3.369; p = < 0.001) higher risk of ADL limitation than their female counterparts, while men had 1.29 times (aOR: 1.29; CI: 1.270–1.930; p = < 0.001) higher odds of ADL limitation.

**Table 3:** Results of logistic regression with confidence interval showing estimates of ADL limitations among older adult?s parents ?left-behind? by their migrant children, India, LASI (2017-18)

Background characteristics	Model 1 Total				Model 2 Male				Model 3 Female			
	UOR	95% CI	aOR	95% CI	UOR	95% CI	aOR	95% CI	UOR	95% CI	aOR	95% CI
Age												
60-69			0.43**	0.348-0.549			0.44**	0.315-0.618			0.40**	0.293-0.560
70-79			0.69**	0.552-0.873			0.63**	0.452-0.880			0.72*	0.527-1.007
80+^@^												
Year of schooling												
0^@^												
1-5			1.07	0.885-1.313			1.07	0.782-1.464			1.13	0.868-1.485
6-9			1.01	0.806-1.282			0.99	0.716-1.386			1.13	0.790-1.642
10+			0.64**	0.510-0.815			0.69**	0.496-0.965			0.67*	0.455-0.992
Currently working status												
Currently not working^@^												
Currently working			0.42**	0.339-0.522			0.40**	0.306-0.525			0.45**	0.309-0.660
Marital status												
Currently in union^@^												
Not in union			1.18*	1.004-1.401			0.98	0.720-1.351			1.16*	0.944-1.443
Living arrangement												
Alone			0.81	0.573-1.164			1.13	0.575-2.224			0.75*	0.495-1.147
Not alone ^@^												
MPCE quintile												
Poorest ^@^												
Poorer			0.80	0.628-1.021			0.84	0.578-1.220			0.77	0.561-1.070
Middle			0.86	0.680-1.094			0.85	0.592-1.236			0.85	0.621-1.164
Richer			0.92	0.725-1.168			0.82	0.567-1.190			0.96	0.702-1.319
Richest			0.87	0.691-1.114			0.92	0.641-1.332			0.80	0.587-1.110
Religion												
Hindu ^@^												
Muslim			1.17	0.978-0.985			0.95	0.687-1.335			1.36**	1.047-1.790
Christian			1.06	1.054-1.071			1.15	0.784-1.693			0.97	0.665-1.418
Others			0.63*	0.396-0.404			0.43**	0.229-0.825			0.84	0.507-1.406
Place of residence												
Rural			1.01	0.859-1.180			1.26*	0.990-1.62			0.88	0.715-1.094
Urban ^@^												
Trust												
Lack of trust	1.05	0.813-1.370	0.96	0.737-1.26	0.96	0.636-1.478	0.95	0.616-1.490	1.05	0.758-1.478	0.98	0.697-1.396
Having trust on others												
Social participation												
No participation	3.22**	2.511-4.132	2.44**	1.882-3.171	3.82**	2.602-5.630	2.96**	1.974-4.444	2.84**	2.050-3.936	2.17**	1.538-3.062
Having participation ^@^												
Friend												
No friend	0.87	0.329-2.312	0.95	0.346-2.118	0.97	0.232-4.134	1.11*	0.252-4.921	0.83	0.218-3.160	0.844	0.219-3.249
Having friends ^@^												
Meeting with friend												
Did not meeting frequency	1.17	0.443-3.117	0.95	0.345-2.612	1,03	0.247-4.320	0.83	0.189-3.660	1.16*	0.301-2.526	1.03	0.263-3.249
Having meeting ^@^												
Talking with friends												
Did not talking	1.77**	1.326-2.379	1.55**	1.142-2.118	1.46*	1.001-2.138	1.29**	1.270-1.930	2.16**	1.327-3.522	2.02**	1.212-3.369
Having talking with friends^@^												

Note- UOR: Unadjusted Odd Ratio; aOR: Adjusted Odd Ratio; @: reference category; ** if p= <0.001; * p=≤0.05; CI= 95% confidence interval

Table-4 Bivariate differences for IADL limitations and social capital among the older adult’s parents ‘left-behind’ by their migrant children, India, LASI (2017-18)

### Social capital and IADL

Table 4 depicts the bivariate difference in IADL limitation and social capital of elder parents with adult migrant sons. It is observed that there is a considerable gender gap in different forms of social capital and IADL limitation among older parents. IADL limitations were present in 32.08% of older males with regard to trust, 30.76% social participation, 26.08% close friends, and 26.01% meeting with friends. These differences were significant (p = < 0.001) as compared to 49.89%, 48.65%, 44.02%, and 43.99% in the respective groups for female counterparts.

**Table 4:** Bivariate differences for IADL limitations and social capital among the older adult?s parents ?left-behind? by their migrant children, India, LASI (2017-18)

Variables	Oder parents having migrant son			
	Male	Female	Z value	P value
Trust				
No trust	31.29	47.94	-1.86	0.061
Have Trust	32.08	49.89	-7.41	<0.001
Social participation				
No participation	56.90	63.19	-0.81	0.414
Any participation	30.76	48.65	7.39	<0.001
Friend				
None	35.23	51.16	-5.92	<0.001
Have friends	26.08	44.02	-3.90	<0.001
Meeting with friends				
Have no meeting	35,20	51.11	-5.93	<0.001
Have any meeting	26.01	43.99	-3.85	<0.001
Talking with friends				
Have no talking	34.55	51.34	-6.85	<0.001
Have any talking	21.32	30.37	-1.24	0.209
Total	33.97	54.13	-9.01	<0.001

Table 5 represents the relationship between several forms of social capital and IADL limitation among older people with at least one migrant son. According to the results of the study, social capital in the forms of social participation and conversation with close friend played a significant role in reducing IADL limitations among elderly parents. There was also a significant gender gap in the association between different forms of social capital and IADL limitation. For the older parent, social participation and social networking in the intensity of meeting and talking with friends played a significant role in IADL limitation. Older men who did not participate in social activities had 1.86 times (aOR: 1.86; CI: 1.237–2.797; P = < 0.001), and did not talk with a friend had 1.20 times (aOR: 1.20; CI: 0.856-1.700; p = < 0.001) higher IADL limitation than their male counterparts, whereas females had 1.09 times (aOR: 1.09; CI: 0.766–1.559; p = < 0.001) more, and 1.74 times (aOR: 1.74; CI: 1.161–2.633; p = < 0.001) respectively IADL limitation than their female counterparts.

**Table 5:** Results of logistic regression with confidence interval showing estimates of IADL limitations among older adult?s parents ?left-behind? by their migrant children, India, LASI (2017-18)

Background characteristics	Model 1 Total				Model 2 Male				Model 3 Female			
	UOR	95% CI	aOR	95% CI	UOR	95% CI	aOR	95% CI	UOR	95% CI	aOR	95% CI
Age												
60-69			0.33**	0.269-0.424			0.33**	0.246-0.460			0.27**	0.191-0.384
70-79			0.53**	0.427-0.679			0.51**	0.378-0.709			0.48**	0.338-0.693
80+^@^												
Year of schooling												
0^@^												
1-5			0.73**	0.614-0.867			0.79	0.605-1.032			0.80*	0.636-1.030
6-9			0.63**	0.524-1.780			0.74*	0.568-0.989			0.67**	0.490-0.933
10+			0.32**	0.261-0.391			0.36**	0.277-0.490			0.36**	0.264-0.510
Currently working status												
Currently not working^@^												
Currently working			0.51**	0.435-0.610			0.48**	0.393-0.607			0.61**	0.463-0.822
Marital status												
Currently in union^@^												
Not in union			1.32**	1.144-1.538			1.14	0.867-1.500			1.21*	1.004-1.462
Living arrangement												
Alone			0.88	0.647-1.198			0.78	0.420-1.455			0.90	0.626-1.296
Not alone ^@^												
MPCE quintile												
Poorest ^@^												
Poorer			0.97	0.788-1.202			1.00	0.732-1.370			0.95	0.719-1.275
Middle			0.87	0.708-1.077			0.71*	0.519-0.983			0.99	0.748-1.318
Richer			0.93	0.760-1.158			0.88	0.643-1.208			0.95	0.714-1.269
Richest			0.91	0.742-1.130			0.93	0.680-1.279			0.84	0.637-1.125
Religion												
Hindu ^@^												
Muslim			1.05	0.876-1.269			0.97	0.737-1.295			1.19	0.924-1.532
Christian			0.95	0.756-1.209			0.80	0.561-1.147			1.07	0.777-1.474
Others			0.70*	0.515-0.972			0.86	0.549-1.362			0.61**	0.397-0.959
Place of residence												
Rural			1.15*	1.009-1.332			1.25*	1.019-1.551			1.14*	0.942-0.961
Urban ^@^												
Trust												
Lack of trust	0.86	0.688-1.087	0.72**	0.566-0.924	0.85	0.591-1.233	0.80	0.543-1.188	0.84	0.628-1.140	0.69*	0.509-0.961
Having trust on others												
Social participation												
No participation	2.09**	1.629-2.687	1.21**	1.034-1.766	2.67**	1.828-3.927	1.86**	1.237-2.797	1.72**	1.239-2.408	1.09**	0.766-1.559
Having participation ^@^												
Friend												
No friend	0.92	0.407-2.103	0.92	0.387-2.201	0.83	0.264-2.614	0.87	0.253-3.032	1.09	0.330-3.640	1.10	0.328-3.708
Having friends ^@^												
Meeting with friend												
Did not meeting frequency	1.18	0.407-2.103	1.05	0.442-2.529	1.33	0.431-4.164	1.18	0.346-4.07	0.84*	0.250-2.846	0.79	0.233-2.733
Having meeting ^@^												
Talking with friends												
Did not talking	2.18**	1.718-2.768	1.41**	1.090-1.826	1.69**	1.233-2.326	1.20**	0.856-1.700	2.50**	1.708-3.671	1.74**	1.161-2.633
Having talking with friends^@^												

Note- UOR: Unadjusted Odd Ratio; aOR: Adjusted Odd Ratio; @: reference category; ** if p= <0.001; * p=≤0.05; CI= 95% confidence interval

### Social capital and probable major depression

The bivariate difference between probable major depression and various forms of social capital is shown in Table 6. According to the findings, different types of social capital played a significant role in probable major depression among older parents with at least one live migrant son. Furthermore, there was also a gender difference in social capital and depression in older parents. Male parents had lower rates of depression than female parents (8.26% vs. 11.32%). Even mothers who trust people had a depression level of 8.46%, 8.30% who participate in social activities, 7.97% who have a close friend, and 7.74% who meet with friends. In contrast, the male prevalence was 6.28%, 5.89%, 5.44%, and 5.53%, accordingly.

**Table 6:** Bivariate differences for major depression and social capital among the older adult’s parents ‘left-behind’ by their migrant children, India, LASI (2017-18)

Variables	Oder parents having migrant son			
Trust	Male	Female	Z value	P value
No trust	4.08	9.79	-3.34	0.0008
Have Trust	6.28	8.46	-4.49	<0.001
Social participation				
No participation	10.34	11.66	-0.11	0.9094
Any participation	5.89	8.30	-5.29	<0.001
Friend				
None	6.51	8.68	-4.16	<0.001
Have friends	5.44	7.97	-4.66	<0.001
Meeting with friends				
Have no meeting	6.45	8.70	-4.32	<0.001
Have any meeting	5.53	7.74	-2.09	0.0357
Talking with friends				
Have no talking	6.67	8.96	-5.09	<0.001
Have any talking	3.85	3.14	-1.95	0.0502
Total	8.26	11.32	13.8	<0.001

Table 7 demonstrates that the relationship between various forms of social capital and probable major depression among older parents with adult male migrated children. The study showed that social capital significantly affected probable major depression. There was a significant gender difference in the relationship between different kinds of social capital and probable major depression. Older men who did not participate in any social activities had 1.75 times (aOR: 1.51; CI: 0.914–3.384; P:<0.05) higher odds of probable major depression than those who participated, and those who did not talk with close friends had 1.74 times (aOR: 1.74; CI: 0.913–3.335; P:<0.05) higher odds of probable major depression. On the contrary, not meeting a friend increased the 2.36 times (aOR:2.36; CI:1. 1.464–3.996; P:<0.05) probability of probable major depression, whereas did not talk with close friends had 2.52 times (aOR:2.36; CI:1. 1.012–3.318; P:<0.001) among females compared to their counterparts. Talking with a friend had a significant effect on women’s and men’s probable major depression.

**Table 7:** Results of logistic regression with confidence interval showing estimates of major depression among older adult?s parents ?left-behind? by their migrant children, India, LASI (2017-18)

Background characteristics	Model 1 Total				Model 2 Male				Model 3 Female			
	UOR	95% CI	aOR	95% CI	UOR	95% CI	aOR	95% CI	UOR	95% CI	aOR	95% CI
Age												
60-69			2.01**	1.287-3.161			2.34**	1.157-4.755			1.60	0.884-2.901
70-79			1.49*	0.937-2.367			1.38	0.666-2.894			1.53	0.836-2.798
80+^@^												
Year of schooling												
0^@^												
1-5			1.07	0.787-1.467			1.12	0.676-1.878			1.24	0.817-1.909
6-9			1.01	0.703-1.467			1.27	0.765-2.111			0.92	0.474-1.797
10+			0.97	0.681-1.400			1.03	0.621-1.732			1.07	0.566-2.0442
Currently working status												
Currently not working^@^												
Currently working			0.80*	0.598-1.078			0.75	0.513-1.119			0.96	0.605-1.554
Marital status												
Currently in union^@^												
Not in union			1.58**	1.226-1.987			1.52*	0.187-2.237			1.46*	1.060-2.026
Living arrangement												
Alone			1.22*	0.760-1.987			0.64	0.187-2.237			1.44	0.844-2.474
Not alone ^@^												
MPCE quintile												
Poorest ^@^												
Poorer			0.86	0.605-1.236			0.78	0.417-1.469			0.92	0.592-1.430
Middle			0.81	0.569-1.173			1.29	0.723-2.300			0.58**	0.363-0.948
Richer			0.82	0.574-1.193			1.15	0.639-2.100			0.63*	0.391-1.018
Richest			0.89	0.629-1.287			1.30	0.725-2.336			0.67	0.421-1.072
Religion												
Hindu ^@^												
Muslim			1.28	0.943-1.742			0.88	0.515-1.513			1.76**	1.198-2.592
Christian			0.49*	0.287-0.850			0.34**	0.138-0.882			0.61	0.309-1.225
Others			0.65	0.338-1.256			0.12**	0.016-0.884			1.32*	0.641-2.742
Place of residence												
Rural			1.81**	1.389-2.363			1.52*	1.021-2.289			2.12**	1.477-3.0511
Urban ^@^												
Trust												
Lack of trust	0.93	0.609-1.426	0.84	0.548-1.316	0.59	0.255-1.369	0.59	0.251-1.391	1.12*	0.681-1.856	1.01	0.605-1.709
Having trust on others												
Social participation												
No participation	1.56**	1.057-2.327	1.51*	1.005-2.274	1.76*	1.393-3.322	1.75*	0.914-3.384	1.43*	0.867-2.388	1.38*	0.812-2.354
Having participation ^@^												
Friend												
No friend	0.65	0.153-2.779	0.63	0.145-2.761	4.17	0.095-182.6	5.28	0.071-39.57	0.38	0.081-1.815	0.32	0.065-1.575
Having friends ^@^												
Meeting with friend												
Did not meeting frequency	1.26	0.294-5.407	1.28	0.291-5.617	0.20	0.095-8.893	1.16	0.002-12.51	1.99*	0.408-9.685	2.36*	1.464-3.996
Having meeting ^@^												
Talking with friends												
Did not talking	2.58**	1.580-3.232	1.99**	1.199-3.330	1.96**	1.055-3.666	1.74*	0.913-3.335	3.66**	1.503-8.927	2.52**	1.012-3.318
Having talking with friends^@^												

Note- UOR: Unadjusted Odd Ratio; aOR: Adjusted Odd Ratio; @: reference category; ** if p= <0.001; * p=≤0.05; CI= 95% confidence interval

## Discussion

With India’s demographic transition and rising urbanization, the number of adult migrants has increased, while the number of older parents ‘left behind’ has also increased. As a result, research into the relationship between the health outcomes of the ‘left behind’ older parents and various forms of social capital in the Indian context is becoming increasingly significant. Using cross-sectional data from the Longitudinal Ageing Study in India (LASI), this study aims to explore the association between different types of social capital and health outcomes of older parents from a gendered perspective. The association of various forms of social capital on the health outcomes of ‘left behind’ older parents were investigated in this study.

Participation in social activities, having close friends, meeting and conversing with friends significantly affected the health outcomes of older parents with at least one living migrant male child. The older parents were found to have better physical health when they participated in social activities, which was also corroborated in earlier studies [41]. Socially active older persons are typically active, whereas those who report poor physical health are less likely to participate in social activities. Furthermore, both males and females participated in social activities, but men’s participation in social activities had a more significant impact on physical health because males are more likely to engage in outdoor activities while females are more likely to engage in indoor activities [65]. Women are increasingly becoming less visible in work participation and other outdoor activities in India [66]. Moreover, studies reported that women in India who are working or socially more active have higher cognitive abilities than their counterparts [67]. Men’s social interaction is marked by more vital instrumental values that lead to materialistic advantages than women’s, which explains why it has a higher impact on men’s physical health than their counterparts [68]. In both types of physical health (i.e., ADL & IADL) of elder parents, the social network has a significant influence. Meeting and chatting with friends lowered ADL and IADL limitations; however, network size in the form of close friends could only lessen ADL limitations in males and did not affect females. Having close friends in the form of a network reduced ADL limitation in male older adults to some extent. However, it did not affect both categories of physical health in female older adults and IADL limitation in male older adults. Similar findings were also reported in research conducted in China [65] and Japan [69]. It has also been observed that social bonds such as groups formed during social volunteering and other socializing activities in urban green spaces like park, promote regular physical activities and consequently boost older adults’ psychological health and social support [70, 71].

Individual form of social capital has little impact on the psychological well-being of elderly persons in India who have been ‘left behind.’ Networking with friends through phone/email conversations substantially impacts the lowering of depression in older parents. When we looked at the various forms of social capital and its effect on ‘left behind’ depressed older parents from the gender perspective, the study found that social capital significantly impacted depression in both males and females. Trust indicates self-assurance and good psychological intention from others [54], and it reduces depression and promotes psychological well-being in older female adults. In a similar line, we also found that females who had a higher level of trust in family members and others had lesser levels of depression. Frequent meeting with close trusted acquaintances and relatives had lowered their level of depression. However, studies reported that relations could negatively impact on the left-behind parents because bad relationships among household members like sons and in-laws may lead to tensions, disputes and negative interaction patterns [72]. Depression is reduced in older male parents who participate in social activities and maintain conversation with close friends. In Indian patriarchal society, elderly males engage themselves in multiple activities like playing cards in groups, attending political or community meetings like caste or religious group meetings, functions or events such as prayers/satsang/bhajan and enjoy higher respect in the community. In contrast, the elderly females mostly confined to home, look after the grandchildren, perform household chores. In addition, when male members were going outside or absent in the home, females had to take care of the household duties. Moreover, making new acquaintances and joining new networks becomes more challenging for females than males. Due to decreasing physical mobility elderly people also tend to lose contact with their existing social networks. Thus, the positive effect of social participation is not reflected among the females compared to males. This is also similar to the findings of the previous study, where direct engagement with friends improves interactive relationships and creates a feeling of connectedness, reducing depression [41]. Our findings strongly support that having a close friend, chatting with friends over the phone/mail and social participation among males were substantially associated with improved psychological and physical well-being. As mentioned in existing literature, adult-child migration is one of the most important reasons for adverse health outcomes (poor mental and physical health outcomes, poor quality of life) of ‘left behind’ parents. In the opposite vein, studies have also shown a beneficial effect of remittances from migrant children on their parents. However, our study shows that the presence of social capital among the ‘left behind’ older adults plays a crucial role in balancing the health outcomes and well-being among the left-behind parents. The study highlights that participation in social activities and maintaining strong social networks, especially among older men, is crucial in enhancing physical health. In contrast, trust, meeting with close friends, and close social interactions are vital in lowering depression among older females.

According to the findings of this study, several interventions can help elder parents in India enhance their stock of various forms of social capital. First, some initiatives like ‘senior centres’ [41] or senior citizen clubs must be taken to expand the stock of social capital. This can increase ‘left behind’ older parents’ participation in social activities, in turn will also benefit their health outcomes. Second, NGO/Volunteer groups working on these issues should be promoted in rural areas to increase the collective activities among the elderly parents who have been ‘left behind.’ Third, the adult offspring of ‘left-behind’ parents should be incited to provide material, emotional support and care for their elderly parents. Furthermore, a healthy neighbourhood relationship should be promoted at the local level, which will increase older adults’ social interaction, trust, and mutual relationship with neighbours, thus improving their psychological well-being and health, particularly among older women.

This study has several drawbacks. First, it was conducted using cross-sectional data, so the association and findings found in this study between different types of social capital and health outcomes cannot be claimed to be causative. Activities of daily living (ADL) and instrumental activities of daily living (IADL) constraints and depression may affect how people perceive and report their self-assessed levels of social capital. So, in the near future based on panel data, a causative study is needed. Second, Indian society is highly stratified in terms of caste, and religion; each state or region has distinctive characteristics. This study was conducted at the macro level, which provided an overall view of the country; a micro-level study should be conducted to understand the relationship better. Furthermore, qualitative studies on this topic are required to comprehend the in-depth relationship between different forms of social capital and the health outcomes of the ‘left behind’ older persons. Additional factors may significantly impact the levels of depression and health outcomes of elderly parents who are left behind but are not included in the study.

## Conclusion

This study examines the relationship between several aspects of social capital, health and well-being among India’s ‘left behind’ older parents, and the gender gap in this relationship. The study concluded that substantial social capital in terms of social network, trust, and social participation improves health outcomes among the elderly. Furthermore, a significant gender gap was observed in this association. In future, the changing social structure and social support of the elderly due to growing urbanization will be a crucial concern. As a result, various measures must be implemented to encourage and grow social participation and social networks. Initiatives such as building older adults’ groups for social volunteering and creating spaces for social interaction like parks and reading clubs in villages and small towns may strengthen social connections among older adults. According to the findings of the study, interventions can aid in developing of trust between elderly parents and their family/friends/neighbours, and some centres of interaction like senior citizen clubs or senior centres should be created to expand the stock of social capital among left-behind parents. This will increase older parents’ participation in social activities and benefit their health outcomes.

## Data Availability

The study is based on secondary data source, is freely available in the public domain through https://www.iipsindia.ac.in/lasi.

## Abbreviations

ADL: Activities of Daily Living
CEB: Census Enumeration Block
IADL: Instrumental Activities of Daily Living
LASI: Longitudinal Ageing Study of India
MPCE: Monthly Per capita Consumption Expenditure
